# Supplementary material for: Assessing population exposure to coastal flooding due to sea level rise
Source: Nat Commun. 2021 Nov 25;12:6900. doi: 10.1038/s41467-021-27260-1 (PMC8617190; doi:10.1038/s41467-021-27260-1)
Supplement: Supplementary file 1 — Supplementary Information [file 41467_2021_27260_MOESM1_ESM.pdf]

Supplementary Note 1

Sea Level Rise Population Exposure Literature Review

We conducted a systematic review of the literature related to risk assessments of sea level rise. We examined articles through the Web of Science's SCI-Expanded and SSCI indices for the following topics:

> TS = (sea-level rise OR sea level rise)
>
> AND TS = (population)
>
> AND TS = (risk OR vulnerability OR exposure).

Our search (April 2, 2020) returned 731 results. We filtered the results to include articles of relatively high impact defined subjectively as those with at least four citations per year (n = 152). We reviewed the filtered publications for relevancy and selected only those with a spatial risk assessment on human populations or infrastructure in the authors' methods (n = 46). For these publications, we identified the spatial zone(s) in their analyses of sea level rise risk and summarized them (see supp. Table 1).

Supplementary Table 1

Source	Average Cites/Year	High Tide	<1-yr Flood	1-Yr Flood	10-Yr Flood	100-Yr Flood	1000-Yr Flood	LECZ	Other Zones
Hallegatte et al. (2013)	76.5			1		1			
McGranahan et al. (2007)	69.93							1	
Neumann et al. (2015)	60					1		1	
Hinkel et al. (2014)	47.57			1		1			
Small and Nicholls (2003)	34.11								Near coastal zone
Nicholls et al. (2011)	27.7		1	1					
Hanson et al. (2011)	26.2	1				1			
Ericson et al. (2006)	24.13								Mean sea level
Hauer et al. (2016)	21.4	1							
Jones and O'Neill (2016)	21.2							1	
Nicholls (2004)	19.76			1			1		
Nicholls et al. (1999)	18.68			1	1	1	1		
Koks et al. (2015)	18.17								10000-yr flood
Muis et al. (2015)	12.83				1	1	1		
Tol (2002)	12.05	1							
Nicholls and Tol (2006)	11.53			1			1		
Hauer (2017)	9.5	1							
Wu et al. (2002)	9.16								Index-based approach
Kummu et al. (2016)	8.4							1	
de Moel et al. (2011)	8.1								Levee-breach scenarios
Kleinosky et al. (2007)	7.93					1			
Frazier et al. (2010)	7.36					1			
Silva et al. (2014)	7.29							1	
Emrich and Cutter (2011)	6.7	1				1			
Yin et al. (2012)	6								Mean sea level
Dasgupta et al. (2011)	5.5	1				1			
Barnard et al. (2019)	5.5	1	1			1			20-yr floods; "average daily conditions" flooding; 5 m
Lichter et al. (2011)	5.2							1	
Martinich et al. (2013)	5.13	1							
Wetzel et al. (2012)	5.11	1						1	

Source	Average Cites/Year	High Tide	<1-yr Flood	1-Yr Flood	10-Yr Flood	100-Yr Flood	1000-Yr Flood	LECZ	Other Zones
Gornitz et al. (2001)	4.85				1	1			2, 5, 25, and 50-yr floods
Murali et al. (2013)	4.75	1							
Anderson et al. (2017)	4.75	1							
Brown et al. (2018)	4.67					1			
Felsenstein et al. (2014)	4.57	1				1		1	
Hardy et al. (2017)	4.5	1							
Mimura (1999)	4.32	1							
Curtis and Schneider (2011)	4.3	1						1	
Dasgupta et al. (2014)	4.29				1				
Paprotny and Terefenko (2017)	4.25	1					1		
Lichter and Felsenstein (2012)	4.11	1	1			1		1	50-yr floods; tsunami events
Hardy and Hauer (2018)	4	1							
Xian et al. (2018)	4			1		1			
Mavromatidi et al. (2018)	4	1							
Benassai et al. (2015)	4							1	
Reguero et al. (2015)	4	1				1			El Nino event
TOTAL	NA	20	3	7	4	17	5	11	10

Meter increment studies assumed to be High Tide studies; Studies not previously having a 100-yr or 1000-yr designator but having storm surge included were assumed to be 100-yr floods.
